# Vitrification versus slow freezing for human ovarian tissue cryopreservation: a systematic review and meta-anlaysis

**DOI:** 10.1038/s41598-017-09005-7

**Published:** 2017-08-17

**Authors:** Qingquan Shi, Yidong Xie, Yan Wang, Shangwei Li

**Affiliations:** 1 Department of Reproductive Medical Center, West China Second University Hospital, Sichuan University, Chengdu, 610000 China; 2 Key Laboratory of Birth Defects and Related Disease of Women and Children, West China Second University Hospital, Sichuan University, Chengdu, 610000 China

## Abstract

Vitrification is a well-accepted procedure for cryopreservation of gametes and embryos. Less is known, however, about its performance in preserving ovarian tissue, for which slow freezing is the current convention. Increasing interest is being focused on vitrification, but there are as yet no standard protocols for its use with ovarian tissue. In part, this is because of the variety of cell types and complex nature of ovarian tissue. We performed a meta-analysis of 14 studies that compared vitrification with slow freezing for cryopreservation of ovarian tissue. In the pooled analysis, there was no significant difference between the two methods in terms of the proportion of intact primordial follicles, but vitrification was associated with significantly less DNA damage. Secondary endpoints included the number of stromal cells, significantly higher with vitrification, and primordial follicle density, which did not differ between the two methods. The present meta-analysis suggests that vitrification may be more effective than slow freezing, with less primordial follicular DNA strand breaks and better preservation of stromal cells. These advantages should lead to improved ovarian function after transplantation.

## Introduction

Thanks to improvements in early cancer diagnosis and therapy, survival rates for women with cancer have increased by more than 1.6% during the past 5 years^1, 2^. However, gonadotoxic treatment can induce great damage to ovarian reserve, frequently leading to premature ovarian failure, with loss of both steroidogenic and gametogenic functions. Additionally, the increasing number of women postponing childbearing for social or financial reasons^3, 4^ will likely increase demands for fertility preservation^5^.

There are several methods used to preserve female fertility, including ovarian tissue cryopreservation (OTC) and cryopreservation of embryos and oocytes. At present, cryopreservation of embryos and oocytes is an accepted, clinically established procedure, whereas OTC has not been endorsed by the American Society of Reproductive Medicine and is still considered experimental^6^. Initially, there were only a few case reports. Successful human ovarian transplantation was first reported by Silber *et al*. with cortical-tissue grafting in monozygotic twins who were discordant for premature ovarian failure^7^. Subsequently, Donnez *et al*.^8^ reported what is deemed to be the first human live birth from orthotropic transplantation of frozen human ovarian tissue in 2004, with another successful live birth achieved by Meirow in 2005.^9^. However, it appears now that there is a worldwide live birth rate of over 30 to 70%, with more than 70 babies^10^. In the opinion of many pioneers, there is now enough evidence to support OTC and to stop considering it an experimental or investigational approach^11^.

There are two methods for OTC: slow freezing and vitrification. Slow freezing has been the conventional technique for years, despite reports of extensive loss of the follicular pool and excessive damage to stromal cells^12^. To date, only two live birth has been reported after vitrification of ovarian tissue; all other live births have resulted from slow-frozen ovarian cortex^13, 14^. However, interest in vitrification is increasingly a focus of investigation^13, 15, 16^. Vitrification seems to be an emerging alternative to slow freezing, as it has been successfully applied for preservation of human blastocysts and oocytes, and good results have been reported for ovarian tissue from rodents, domestic animals, and non-human primates^15, 17, 18^. Vitrification prevents the formation of ice crystals, reducing the risk of mechanical injury to cells. Compared with slow freezing, vitrification also has the advantages of being a time-saving, simple process, requiring no special or expensive equipment. It is also reported that vitrification preserves the morphologic integrity of stromal cells better than slow freezing^19–22^. Compared with conventional slow-frozen tissues, Rahimi *et al*.^23^ found a higher percentage of apoptotic cells in vitrified ovarian tissues. Also as the originators of ovarian tissue vitrification, Silber S. compared the viable oocytes in vitrified tissue and slow freezing tissue, suggesting that vitrification might provide better results after transplantation^24^. However, Oktem *et al*. reported lower primordial follicle density and viability after vitrification^25^. In contrast, other studies failed to show significant differences between the two methods in terms of the proportion of morphologically intact follicles and apoptotic cells^26, 27^. Because there is as yet no optimal protocol for vitrification, data on human ovarian tissue vitrification are still limited and conflicting. Thus, whether vitrification is superior to slow freezing for OTC remains under debate. The purpose of the present meta-analysis is to evaluate the efficacy of ovarian tissue vitrification and to seek to identify which is the better method for OTC.

## Results

### Study characteristics

**Figure 1:**
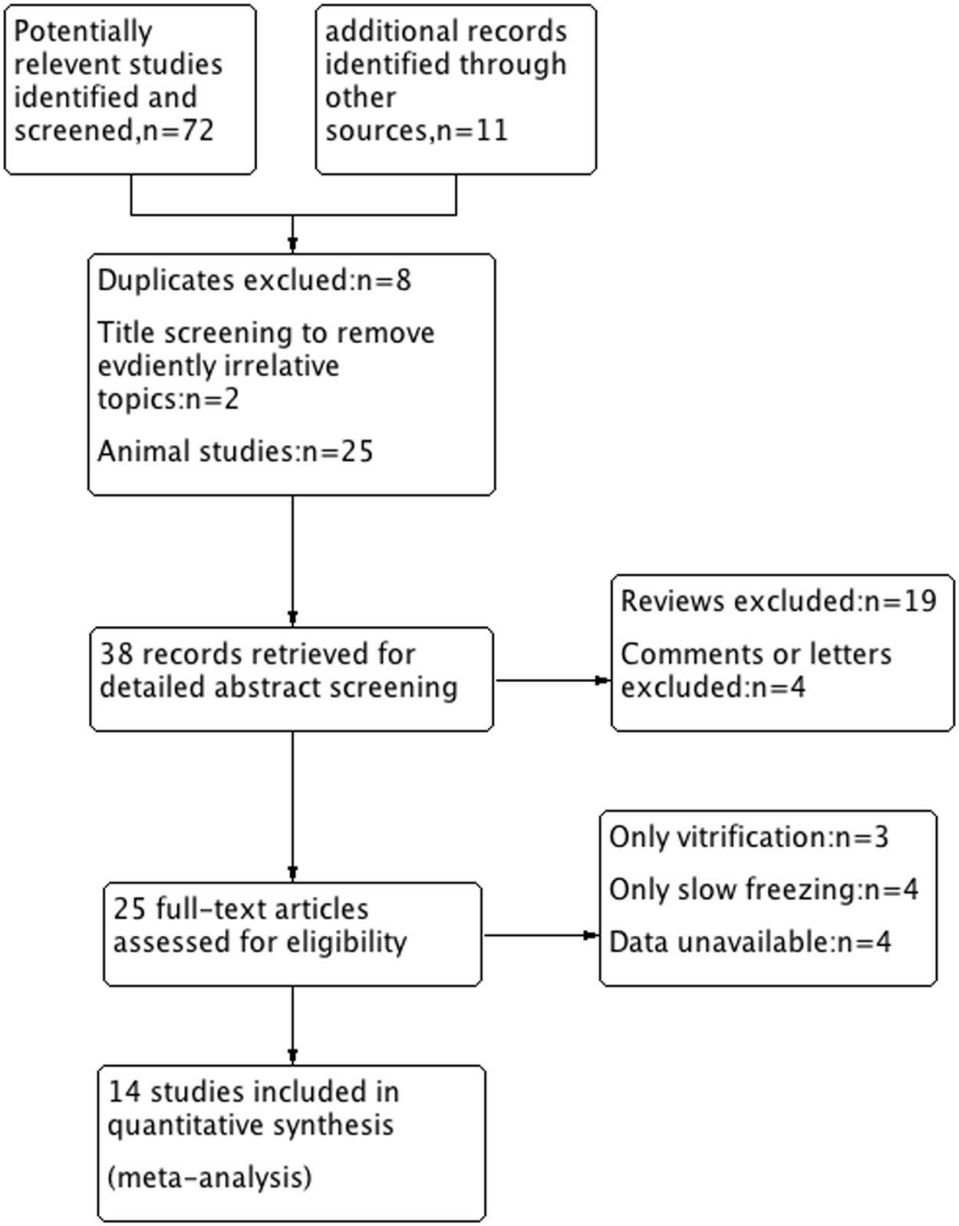
Flow diagram of the study selection process.

The search identified 83 reports, of which 58 were excluded following removal of duplicates and review of titles and abstracts. A further 10 studies were excluded after reading the full text (Fig. 1). Data were extracted from the remaining 14 non-randomized comparative studies 19AQ1^20, 21, 25–35^ and analyzed. The number of study patients ranged from 3 to 26 with ages ranging from 14 to 43 years. Ovarian tissues were retrieved by laparoscopy or laparotomy performed for benign gynecologic conditions or at the time of cesarean section or cancer patients who have oophorectomy, ovarian cystectomy, and ovary transposition. The vitrification protocols vary from tissue carrier and cryoprotectant. The number of primordial follicles ranged from 176 to 1015, among which 134 to 611 were morphologically intact primordial. Additionally, total number of the evaluation of DNA fragmentation in primordial follicles ranged from 56 to 781, among which 22 to 245 were primordial follicle with DNA fragmentation. Overall, included studies that assessed the stromal cells ranged from 56 to 781, among which 22 to 245 were normal stromal cells. A summary of the main characteristics and outcomes of the identified studies is presented in Table 1.

**Table 1:** Basic characteristics and outcomes of studies included.

First author (publication year)	Patients	Age	Vitrification method			primordial follicle		DNA fragmentation evaluation		Evaluation of stromal cells		Outcomes
			ovarian tissue carrier	equilibration solution	vitrification solution	Total	Intact follicle	Total	follicle with DNA fragmentation	Total	normal stromal cells	
Fabbri (2016)	6	Range:14–34	open plastic home-made support	2 M propylene glycol + 3 M EG + 0.2 M Sucrose for 30 mins	3 M propylene glycol + 5 M EG + 0.5 M Sucrose + 15%HS 30 mins	642	352	NA	NA	NA	NA	estimate follicular density, Follicle intact morphology, intracellular ROS localization and levels states of ovarian follicles and stroma
Sanfulippo (2015)	5	Mean: 28 (1.1)	droped dircetly into liquid nitrogen	**step 1**: 0.37 M PrOH + 0.37 M EG for 5 mins **step 2**: 0.75 M PrOH + 0.75 M EG for 5 mins	1.5 M PrOH + 1.5 M EG + 0.5 M raffinose for 10 mins	856	685	93	22	NA	NA	estimate follicular density Follicle intact morphology DNA fragmentation in the follicles by TUNEL
Klocke (2015)	23	Mean: 29.9 (5.0)	acupuncture needle	7.5% EG + 7.5%DMSO + 20%HSA for 15 mins	15% EG + 15% DMSO + 0.5 M sucrose for 2 mins	NA	NA	NA	NA	NA	NA	the proportion of high-quality follicles Evaluation of the integrity of the follicles after tissue culture Estradiol production in the ovarian culture system
Herraiz (2014)	8	Mean: 27	metallic grids	7.5% EG + 7.5% DMSO for 25 mins	20% EG + 20% DMSO + 0.5 M sucrose + 20%synthetic serum substitute for 15 mins	1015	526	510	182	NA	NA	follicular densities Morphometric Study Cell Proliferation and Vascularization DNA Strand Breaks by TUNEL fibrotic surface area
Xiao (2013)	3	Range:20–36	acupuncture needle	7.5% EG + 7.5% DMSO for 10 mins	13.5% EG + 13.5% DMSO + 0.5 M sucrose for 2 mins	271	180	294	115	NA	NA	abnormal primordial follicles apoptotic primordial follicles
Amorim (2012)	7	Range: 30–41	**Protocol 1:** Solid-surface **protocol 2:** open cryostraws	**protocol 1**: Step1, 5% DMSO + 5% EG 5 mins step2, 10% DMSO + 10% EG 5 mins **protocol 2**: Step1,2.5% DMSO + 6.5% EG 5 mins step2, 5% DMSO + 13% EG 5 mins	**protocol 1**: 20% DMSO and 20% ethylene glycol 10 mins **protocol 2**: 10% DMSO and 26% ethylene glycol 1 min	287	172	56	14	NA	NA	Morphologically normal follicles Follicular proliferation and apoptosis
Oktem (2011)	15	Range: 18–37	cryovials	15% propanediol + 15% EG + 0.2 M sucrose for 10 mins	NR	NA	NA	NA	NA	NA	NA	Follicle density AMH and estradiol levels in culture fluid
Chang (2011)	11	Mean: 31.9	cryovial	**Group**1, 20% EG for 5 mins; **group2**, 20% EG for 10 min; **group3**: 20% EG for 20 mins	40% EG, 18% Ficoll, and 0.3 M sucrose 5 mins	268	161	270	127	600	319	morphologically intact follicles Detection of apoptotic follicle by TUNEL
Xiao (2010)	10	Range: 21–36	acupuncture needle	7.5%EG + 7.5%DMSO for 10 mins	**Group3**: 2.69 M EG (15%) + 2.11 M DMSO (15%) + 0.5 M sucrose 2 mins **Group3**: 2.42 M EG (13.5%) + 1.90 M DMSO (13.5%) + 0.5 M sucrose 2 mins **Group C**: 2.15 M EG (12%) + 1.69 M DMSO (12%) + 0.5 M sucrose 2 mins	760	611	781	245	800	376	assess morphologic damage of the primordial follicles Oocytes, granulosa cells of primordial follicles, and stromal cells were analyzed using TEMTUNEL Assay for primordial follicle and stromal cells Detection of Apoptosis
Keros (2009)	20	Range: 20–41	Hand-cut cryostraws	**Group 1: Step 1**: 0.35 M DMSO + 0.38 M PrOH + 0.38 M Eg for 5 mins **step 2**: 0.7 M DMSO + 0.75 M PrOH + 0.75 M EG for 5 mins **Group 2**: **Step 1** 0.35 M DMSO + 0.38 M PrOH + 0.38 M EG for 10 mins **step2** : 0.7 M DMSO + 0.75 M PrOH + 0.75 M EG for 10 mins	**Group 1**: 1.4 M DMSO + 1.5 M PrOH + 1.5 M EG + polyvinylpy-rrolidone for 5 mins **Group 2**: 1.4 M DMSO, 1.5 M PrOH, 1.5 M EG + polyvinyl-pyrrolidone for 10 mins	279	144	NA	NA	26763	13513	The number of follicles of different developmental stages was evaluated. intact the follicles proportion of intact stromal cells assessing the structures of oocytes, granulosa cells and the stroma
Wang (2008)	5	Range: 21–37	acupuncture needle	7.5% EG + 7.5% DMSO for 10 mins	15% EG + 15% DMSO + 0.5 M sucrose 2 mins	272	225	NA	NA	400	169	follicle morphology; The oocytes, the granulosa cells of the primordial follicles and the stromal cells surrounding follicles were evaluated separately; Viability assay
			Pasteur pipette.	10% EG + 10% DMSO for 5 mins	20% EG + 20% DMSO 5 mins							
Huang (2008)	26	Mean: 29.9 (3.4)	Solid-surface vitrification	**Step 1**: 5% DMSO + 5% EG for 5 mins **Step 2**: 10% DMSO + 10% EG for 5 mins	**Step 1:** 15%(DMSO) + 15% (EG)10 mins **Step 2:** 20%(DMSO) + 20% (EG)10 mins	623	528	NA	NA	NA	NA	The percentage of morphologically normal primordial follicles; Assessment of apoptosis in primordial follicles and stromal cells; Production of hormones by warmed/thawed ovarian tissue
Li (2007)	15	Mean: 33.1	Minimum drop size directly plunged into LN2	2 M DMSO + O.1 M sucrose for 5 mins	2.M DMSO + 2.M(PROH) + 0.2 M sucrose 5 mins	176	134	NA	NA	NA	NA	Proportions of normal primordial follicle; concentration of estrogen and progesterone
Gandolfi (2006)	3	Range: 26–33	Straws directly plunged into LN2	0.64 M EG + 20% FBS for 30 mins	5.64 M EG + 5% polyvinylpyrrolidone + 0.4 M trehalose for 2 mins	550	277	NA	NA	NA	NA	Normality of follicular structures

EG = ethylene glycol, DMSO = dimethyl sulphoxide, PrOH = 1,2-propanediol, NA = not applicable, NR = not reported, M = mol/L

*Data were presented as range and/or mean (standard deviation).

### Quality assessment

The average Newcastle–Ottawa Quality Assessment Scale score of included studies was 7.07 (range 6–8), which suggested that the majority of eligible studies were of relatively high quality (Table 2). All the articles had clear descriptions of the intervention and measured the outcomes with objective and/or subjective methods. Overall, the results indicated that the studies were of good quality.

**Table 2:** The detail NOS score of studies included.

	Fabbri (2016)	Sanfulippo (2015)	Klocke (2015)	Herraiz (2014)	Xiao (2013)	Amorim (2012)	Oktem (2011)	Chang (2011)	Xiao (2010)	Keros (2009)	Wang (2008)	Huang (2008)	Li (2007)	Gandolfi (2006)
Were the exposed cohorts somewhat representative in the community?	yes	yes	yes	yes	yes	yes	yes	yes	yes	yes	yes	yes	yes	yes
Were the non exposed cohorts drawn from the same community as the exposed cohorts?	yes	yes	yes	yes	yes	yes	yes	yes	yes	yes	yes	yes	yes	yes
Did the ascertainment of exposure have a secure record?	yes	yes	yes	yes	yes	yes	yes	yes	yes	yes	yes	yes	yes	yes
Demonstration that outcome of interest was not present at start of study?	yes	yes	yes	yes	yes	yes	NA	yes	yes	yes	yes	yes	NA	yes
Was the intervention clearly described in the study?	yes	yes	yes	yes	yes	yes	yes	yes	yes	yes	yes	yes	yes	yes
Were additional interventions (co-interventions) clearly reported in the study?	yes	NA	no	no	yes	no	NA	yes	yes	yes	yes	yes	NA	yes
Were relevant outcomes appropriately measured with objective and/or subjective methods?	yes	yes	yes	yes	yes	yes	yes	yes	yes	yes	yes	yes	yes	yes
Was follow-up long enough for outcomes to occur?	yes	yes	yes	yes	yes	yes	yes	yes	yes	yes	yes	yes	yes	yes
Was the loss to follow-up reported?	NA	NA	NA	NA	NA	NA	NA	NA	NA	NA	NA	NA	NA	NA
NOS score	8	7	7	7	8	7	6	7	7	7	8	7	6	7

NA = not applicable

### Proportion of intact primordial follicles

**Figure 2:**
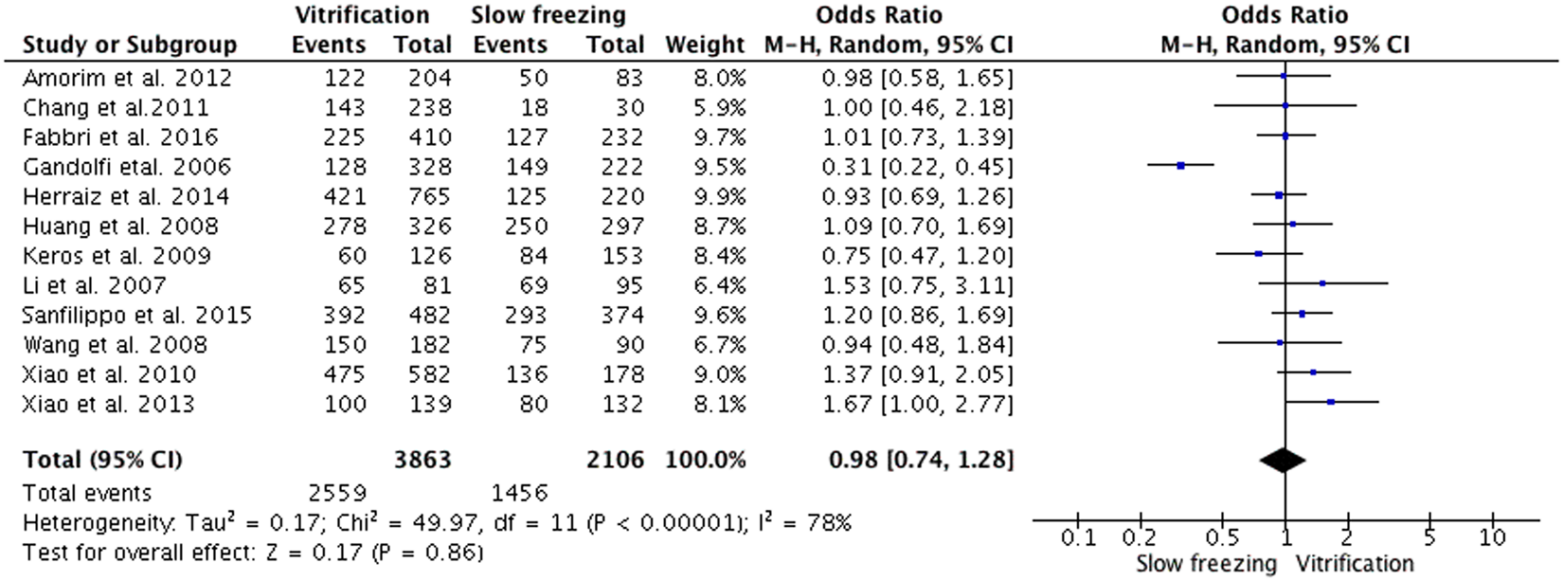
Random effect model of odds ratio with 95% CIs of the proportion of intact primordial follicles: slow freezing versus vitrification.

Intact primordial follicles were measured in 12 studies, of which two^28, 33^ indicated that vitrification was associated with a significantly higher proportion of intact primordial follicles compared with slow freezing. The remaining 10 studies did not observe any differences in the number of intact primordial follicles between the two methods. There was significant evidence of heterogeneity across the 12 studies (I^2^ = 69%), therefore, a random effect model was used for the pooled estimates. The pooled OR showed no significant difference in the proportion of intact primordial follicles after slow freezing or vitrification (OR = 0.98; 95% CI, 0.74–1.28; P = 0.86; Fig. 2)

### DNA fragmentation in primordial follicles

**Figure 3:**
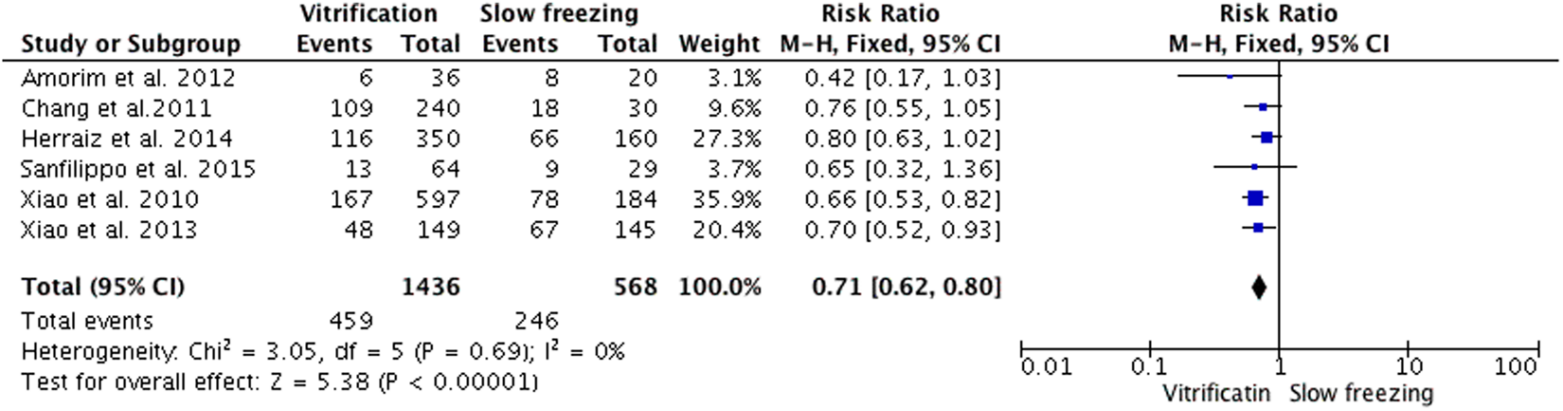
Fixed model of risk ratio with 95% CIs of the DNA fragmentation in primordial follicles: vitrification versus slow freezing.

Six studies reported DNA strand breaks in primordial follicles. Two studies by Xiao^20, 33^ showed that vitrification was associated with significantly less DNA fragmentation in primordial follicles compared with slow freezing. However, the remaining four trials did not show any differences in terms of follicular DNA damage. Overall, the pooled analysis of these studies revealed significantly less DNA damage in primordial follicles with vitrification than with slow freezing (RR = 0.71; 95% CI, 0.62–0.80; P < 0.00001; Fig. 3).

### Proportion of normal stromal cells

**Figure 4:**
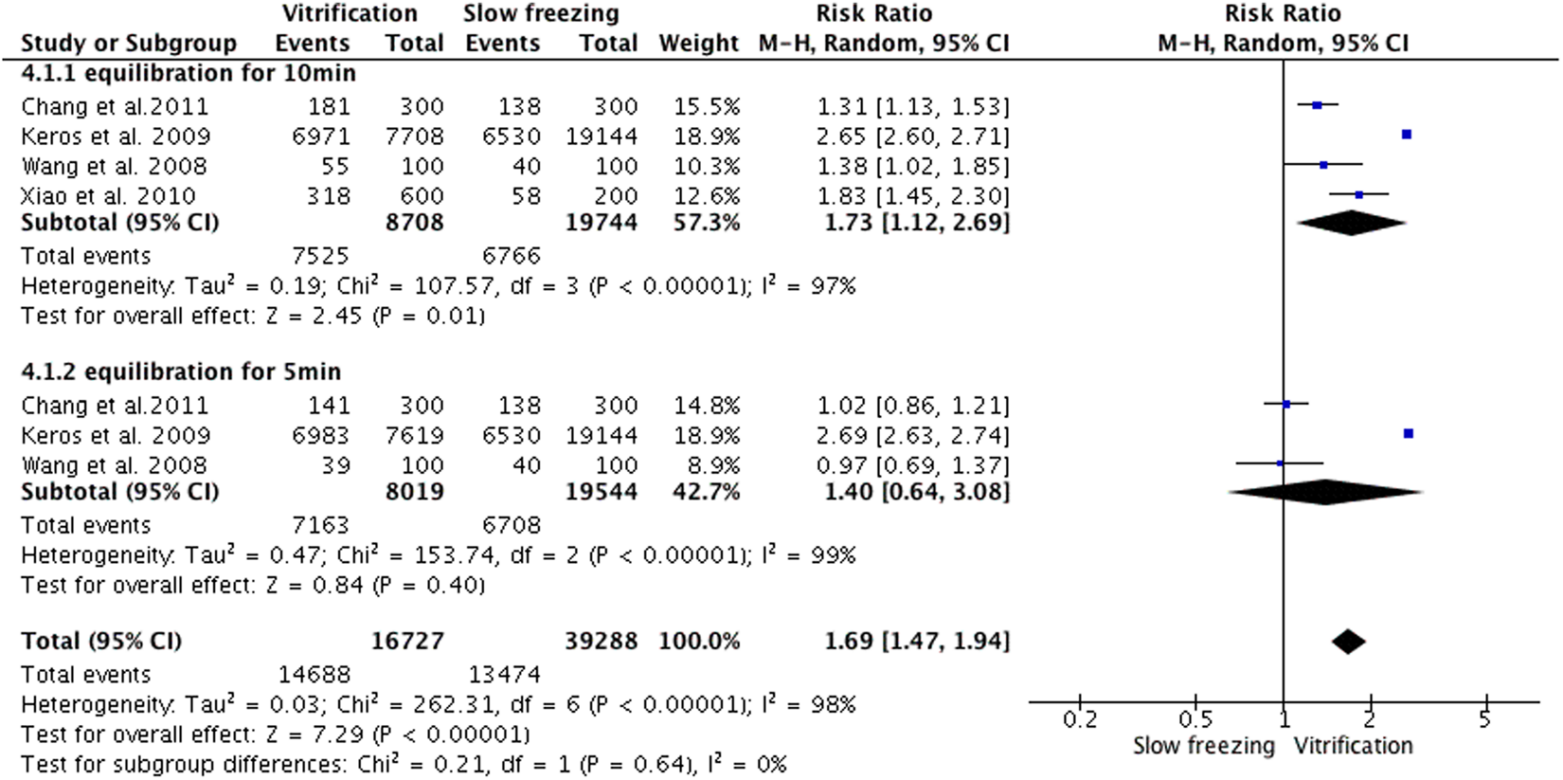
Random effect model of risk ratio with 95% CIs of the proportion of normal stromal cells: slow freezing versus vitrification.

Six studies evaluated stromal cells, but adequate data were available in only 4^19–21, 35^. When ovarian tissues were pretreated with equilibration solution for 10 minutes before vitrification, pooled analysis showed that vitrification protected more stromal cells than did slow freezing. However, if ovarian tissues were pretreated for 5 minutes, there was no significant difference in the proportion of normal stromal cells between the two methods. Overall, the pooled subgroup analysis of these studies revealed significantly more normal stromal cells after vitrification than after slow freezing (RR = 1.69; 95% CI, 1.47–1.94; P < 0.00001; Fig. 4).

### Primordial follicle density

**Figure 5:**
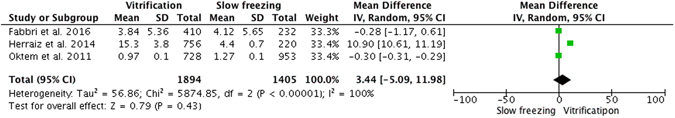
Random effect model of the mean difference with 95% CIs of the primordial follicle density: slow freezing versus vitrification.

Only three studies reported data on primordial follicle density^25, 29, 35^. Herraiz indicated the vitrification was associated with significantly greater primordial follicle density than slow freezing^32^. However, the other two studies did not find any difference^25, 29^. The pooled analysis revealed no significant differences in primordial follicle density between vitrification and slow freezing (IV = 3.44; 95% CI, −5.09–11.98; P = 0.43; Fig. 5).

## Disscussion

Due its excellent outcomes, vitrification has replaced conventional slow freezing as the primary approach to cryopreservation of gametes and embryos^17, 21, 36^. However, since the ovary is an organ composed of diverse cell types, OTC is more complicated. Additionally, the outcomes of cryopreservation depend on multiple factors including the cryoprotectants, the size of ovarian fragments, and the speed of cooling^15, 37^. OTC requires balancing of effects on the oocytes, granular cells, and stroma. Whether vitrification is a better procedure for preserving ovarian fragments is still controversial, so vitrification and slow freezing have been used recently to investigate its effects on stromal cells, as well as the morphologic integrity of primordial follicles. Early studies indicated that the slow-frozen ovarian cortex was better preserved than vitrified ovarian tissue^28^. However, vitrification has more recently been suggested to have positive effects on granulosa cells and ovarian stroma, providing equivalent or better results than slow cooling for preserving ovarian tissue^19, 21, 27, 32, 33, 38^. The purpose of this meta-analysis of 14 studies was to evaluate the efficacy of ovarian tissue vitrification compared with slow freezing, including evaluation of stromal cells as a secondary outcome, and to identify which method is better for OTC. And we find vitrification may be more effective than slow freezing, with less primordial follicular DNA strand breaks and better preservation of stromal cells.

In the frozen ovarian cortex, the appearance and quality of follicles may in part predict the possibility of restoring reproductive function. Primordial follicles are the primary type in cryopreserved human ovarian fragments, accounting for more than 90% of all follicles. Thus, when it comes to assessing the efficacy of cryopreservation, a common endpoint is morphologic intactness of primordial follicles. Such investigations were performed by Gandolfi *et al*. on human, bovine, and porcine ovarian tissues. They concluded that conventional freezing results in much better preservation of all follicle types than does vitrification^28^. Conversely, Xiao *et al*. reported that a novel vitrification technique was comparable to slow freezing with respect to preserving primordial follicles in human ovarian tissue. The percentage of morphologically abnormal primordial follicles was significantly lower with vitrification than with slow freezing^33^. But Oktem *et al*. found that vitrified human ovaries have fewer primordial follicles and produce less anti-Müllerian hormone than slow-frozen ovaries^25^. In this meta-analysis, 12 studies recorded data on intact primordial follicles, and the overall pooled analysis showed no difference between vitrification and slow freezing for this endpoint. Additionally, the primordial follicle density of ovarian tissue was evaluated in three studies, and no significant difference between the two methods was found.

Although follicles may be morphologically intact after cryopreservation, their ability to develop and produce an oocyte to be fertilized, with the ultimate achievement of pregnancy and live birth, may be affected by cryopreservation, regardless of the freezing method. Apoptosis has been suggested as a marker of the developmental capacity of primordial follicles after cryopreservation^39^. Rimon *et al*. observed that apoptosis was already present in follicles that appeared normal when examined only morphologically^39^. They concluded that apoptosis might be a more definitive indicator of the viability of follicles than the general morphologic appearance. In the investigation by Xiao *et al*., the incidence of TUNEL-positive primordial follicles was lower after vitrification than after slow freezing^33^. Overall, analysis of pooled data in the present review demonstrated that vitrification yielded a lower proportion of DNA fragmentation in primordial follicles.

Vitrification is an ultra-rapid cooling technique with no ice crystal formation. Conversely, the faster cooling rate means a higher concentration of cryoprotectant, which may be toxic to living cells. Thus, a variety of cryoprotectants have been used in combination to reduce the toxicity of individual agents while still achieving a highly viscous solution^15, 19–27^. To date, however, there is still no optimal vitrification protocol. In the studies we reviewed, vitrification was routinely achieved by initial exposure of ovarian tissues to concentrations of permeating cryoprotectants (commonly combinations of ethylene glycol, Dimethyl Sulphoxide, propylene glycol and propyl alcohol) similar to those employed prior to conventional slow cooling. The duration of exposure to the high concentrations before loading onto a carrier differed among the studies.

Since the ovary is an organ composed of heterogeneous cellular components, diffusion rates of the cryoprotectants and the potential for ice crystal formation vary for each cell and tissue type^40^. Thus, OTC involves a compromise between effects on the oocytes, follicular cells, and stroma, the latter comprising blood vessels, nerves, and extracellular matrix. The stromal cells can transform into the theca interna and externa outside the follicular basal lamina, which is believed to positively affect granulosa cell proliferation and differentiation^19^. So, to evaluate the effect of cryopreservation, investigations of stromal cells are as essential as those of the follicles. However, we found only four studies that evaluated stromal cells. These explored the best concentrations of cryoprotectant and exposure times in the vitrification protocol. In those four studies, the protocol was a two-step process including equilibration and vitrification. We therefore performed a subgroup analysis of the effect on stromal cells according to equilibration time. Overall, the pooled subgroup analysis of these studies revealed that vitrification resulted in significantly more normal stromal cells than did slow freezing.

There are limitations of this analysis that need to be considered. The studies included varied terms of in the vitrification procedure and cryoprotectants used. We were unable to examine the effect of different vitrification protocols because of insufficient data. In addition, the outcome was only based on morphologic examination and cell vitality, including DNA strand breaks. We did not have data on the endocrine function of the ovarian fragments. Lastly, we assessed only the primordial follicles, not growing follicles. Since a larger number of primordial follicles does not necessarily mean better results in terms of pregnancy, the potential of these primordial follicles to develop into primary or secondary follicles, mature, and produce oocytes capable of fertilization should also be evaluated. In the future, a different approach to OTC might involve removal of small antral follicles, followed by *in vitro* maturation. Immature oocytes can also be collected from antral follicles in ovarian tissue at the time of cryopreservation, matured *in vitro*, and then cryopreserved to await fertilization^41^.

## Conclusion

Based on the data available, the present meta-analysis suggests that vitrification may be more effective than slow freezing for OTC, resulting in with fewer primordial follicular DNA strand breaks and better preserved stromal cells, which should lead to improved tissue function after transplantation. However, the included studies varied in terms of the vitrification protocol used. These findings must now be validated in prospective randomized studies, with healthy live births as the primary endpoint.

## Methods

### Design and Search Strategy

This systematic review and meta-analysis was conducted in accordance with PRISMA guidelines^42^. A comprehensive and systematic literature search was performed independently by Q.S. and Y.X. Online electronic databases, including PubMed, EMBASE, and the Cochrane database, were searched for studies published from their inception until December 2016. To identify any potentially relevant studies, the following MeSH terms were used: (((((ovarian cortex) OR ovarian tissue) OR ovarian strips) OR whole ovary)) AND ((((((((vitrification) OR ultra-rapid freezing) OR rapid cooling)) OR rapid freezing) OR ultra-rapid cooling)) AND (((((slow cooling) OR slow freeze) OR conventional slow freezing) OR slow-frozen) OR slow cryopreservation)). The references of all identified studies were also searched. Two investigators evaluated all identified trials and separately assessed the methodologic quality of the studies. Any discrepancies were resolved by mutual discussion.

### Inclusion and Exclusion Criteria

The following criteria were used for study selection: 1) studies were randomized controlled trials/cohort/observational studies; 2) comparison of human ovarian tissue which were preserved by vitrification and conventional slow freezing; 3) outcomes included at least one of the following endpoints: morphologically intact primordial follicles/DNA fragmentation in primordial follicles/stromal cell/primordial follicle density.

Exclusion criteria were as follows: 1) letters, comments, editorials, case reports, and reviews; 2) studies with no comparison of vitrification with slow freezing; 3) no quantitative primary or second outcome data reported; 4) animal studies.

### Outcome Measures

The primary outcome measure was the proportion of morphologically intact tissue and DNA fragmentation in primordial follicles after cryopreservation. The secondary outcomes were the proportion of normal stromal cells and primordial follicle density (follicles/mm^2^).

### Study Quality Assessment

For RCTs we will use the risk bias assessment According to the criteria of the Cochrane Handbook for Systematic Reviews of Interventions, for observational/cohort/case-control studies, the Newcastle–Ottawa Quality Assessment Scale, a rating tool that evaluates the quality of non-randomized studies from three perspectives (selection, comparability and outcome), was used to assess the validity^43^. This rating system’s score ranges from 0 to 9, and studies with a score of more than 7 were assumed to be of high quality.

### Data Extraction and Statistical Analysis

Two reviewers extracted the relevant data independently, and these data were then cross-checked. The following information was extracted from each study: study design, year of publication, population characteristics, and relevant outcome data. Any disagreements that could not be reconciled by discussion were considered by a third person (SW.L.). This meta-analysis was performed in accordance with the recommendations of the Cochrane Collaboration^43^. Statistical analyses were conducted using Review Manager 5.1.0. The Mantel-Haenszel χ^2^ test and I^2^ test were used to assess statistical heterogeneity. When the I^2^ value was less than 50%, heterogeneity was considered to be acceptable^43^. A fixed effect model was used for calculations in the absence of evidence of heterogeneity; otherwise, a random effect model was applied. Risk ratios (RR) were used to evaluate dichotomous variables, while mean differences were used to evaluate continuous variables; both were accompanied by 95% confidence intervals (CI). For these trials, a P value < 0.05 was considered to be statistically significant.
